# Data Mining Techniques in Analyzing Process Data: A Didactic

**DOI:** 10.3389/fpsyg.2018.02231

**Published:** 2018-11-23

**Authors:** Xin Qiao, Hong Jiao

**Affiliations:** University of Maryland, College Park, College Park, MD, United States

**Keywords:** data mining, log file, process data, educational assessment, psychometric

## Abstract

Due to increasing use of technology-enhanced educational assessment, data mining methods have been explored to analyse process data in log files from such assessment. However, most studies were limited to one data mining technique under one specific scenario. The current study demonstrates the usage of four frequently used supervised techniques, including Classification and Regression Trees (CART), gradient boosting, random forest, support vector machine (SVM), and two unsupervised methods, Self-organizing Map (SOM) and *k*-means, fitted to one assessment data. The USA sample (*N* = 426) from the 2012 Program for International Student Assessment (PISA) responding to problem-solving items is extracted to demonstrate the methods. After concrete feature generation and feature selection, classifier development procedures are implemented using the illustrated techniques. Results show satisfactory classification accuracy for all the techniques. Suggestions for the selection of classifiers are presented based on the research questions, the interpretability and the simplicity of the classifiers. Interpretations for the results from both supervised and unsupervised learning methods are provided.

## Introduction

With the advance of technology incorporated in educational assessment, researchers have been intrigued by a new type of data, process data, generated from computer-based assessment, or new sources of data, such as keystroke or eye tracking data. Most often, such data, often referred to as “data ocean,” is of very large volume and with few ready-to-use features. How to explore, discover and extract useful information from such an ocean has been challenging.

What analyses should be performed on such process data? Even though specific analytic methods are to be used for different data sources with specific features, some common analysis methods can be performed based on the generic characteristics of log files. Hao et al. (2016) have summarized several common analytic actions when introducing the package in Python, glassPy. These include summary information about the log file, such as the number of sessions, the time duration of each session, and the frequency of each event, can be obtained through a summary function. In addition, event n-grams, or event sequences of different lengths, can be formed for further utilization of similarity measures to classify and compare persons' performances. To take the temporal information into account, hierarchical vectorization of the rank ordered time intervals and the time interval distribution of event pairs were also introduced. In addition to these common analytic techniques, other existing data analytic methods for process data are Social Network Analysis (SNA; Zhu et al., 2016), Bayesian Networks/Bayes nets (BNs; Levy, 2014), Hidden Markov Model (Jeong et al., 2010), Markov Item Response Theory (Shu et al., 2017), diagraphs (DiCerbo et al., 2011) and process mining (Howard et al., 2010). Further, modern data mining techniques, including cluster analysis, decision trees, and artificial neural networks, have been used to reveal useful information about students' problem-solving strategies in various technology-enhanced assessments (e.g., Soller and Stevens, 2007; Kerr et al., 2011; Gobert et al., 2012).

The focus of the current study is about data mining techniques and this paragraph provides a brief review of related techniques that have been frequently utilized and lessons that have been learned related to analyzing process data in technology-enhanced educational assessment. Two major classes of data mining techniques are supervised and unsupervised learning methods (Fu et al., 2014; Sinharay, 2016). Supervised methods are used when subjects' memberships are known and the purpose is to train a classifier that can precisely classify the subjects into their own category (e.g., score) and then be efficiently generalized to new datasets. Unsupervised methods are utilized when subjects' memberships are unknown and the goal is to categorize the subjects into clearly separate groups based on features that can distinguish them apart. Decision trees, as a supervised data classification method, has been used very often in analysing process data in educational assessment. DiCerbo and Kidwai (2013) used Classification and Regression Tree (CART) methodology to create the classifier to detect a player's goal in a gaming environment. The authors demonstrated the building of the classifier, including feature generation, pruning process, and evaluated the results using precision, recall, Cohen's Kappa and A' (Hanley and McNeil, 1982). This study proved that the CART could be a reliable automated detector and illustrated the process of how to build such a detector with a relative small sample size (*n* = 527). On the other hand, cluster analysis and Self-Organizing Maps (SOMs; Kohonen, 1997) are two well-established unsupervised techniques that categorize students' problem-solving strategies. Kerr et al. (2011) showed that cluster analysis can consistently identify key features in 155 students' performances in log files extracted from an educational gaming and simulation environment called *Save Patch* (Chung et al., 2010), which measures mathematical competence. The authors described how they manipulated the data for the application of clustering algorithms and showed evidence that fuzzy cluster analysis is more appropriate than hard cluster analysis in analyzing log file process data from game/simulation environment. Most importantly, the authors demonstrated how cluster analysis can identify both effective strategies and misconceptions students have with respect to the related construct. Soller and Stevens (2007) showed the power of SOM in terms of pattern recognition. They used SOM to categorize 5284 individual problem-solving performances into 36 different problem-solving strategies, each exhibiting different solution frequencies. The authors noted that the 36 strategy classifications can be used as input to a test-level scoring process or externally validated by associating them with other measures. Such detailed classifications can also serve as valuable feedback to students and instructors. Chapters in Williamson et al. (2006) also discussed extensively the promising future of using data mining techniques, like SOM, as an automated scoring method. Fossey (2017) has evaluated three unsupervised methods, including *k*-means, SOM and Robust Clustering Using Links (ROCK) on analyzing process data in log files from a game-based assessment scenario.

To date, however, no study has demonstrated the utilization of both supervised and unsupervised data mining techniques for the analysis of the same process data. This study aims at filling this gap and provides a didactic of analyzing process data from the 2012 PISA log files retrieved from one of the problem-solving items using both types of data mining methods. This log file is well-structured and representative of what researchers may encounter in complex assessments, thus, suitable for demonstration purposes. The goal of the current study is 3-fold: (1) to demonstrate the use of data mining methods on process data in a systematic way; (2) to evaluate the consistency of the classification results from different data mining techniques, either supervised or unsupervised, with one data file; (3) to illustrate how the results from supervised and unsupervised data mining techniques can be used to deal with psychometric issues and challenges.

The subsequent sections are organized as follows. First, the PISA 2012 public dataset, including participants and the problem-solving item analyzed, is introduced. Second, the data analytic methods used in the current study are elaborated and the concrete classifier development processes are illustrated. Third, the results from data analyses are reported. Lastly, the interpretations of the results, limitations of the current study and future research directions are discussed.

## Methods

### Participants

The USA sample (*N* = 429) was extracted from the 2012 PISA public dataset. Students were from 15 years 3 months old to 16 years 2 months old, representing 15-year-olds in USA (Organisation for Economic Co-operation Development, 2014). Three students with missing student IDs and school IDs were deleted, yielding a sample of 426 students. There were no missing responses. The dataset was randomly partitioned into a training dataset (*n* = 320, 75.12%) and a test dataset (*n* = 106, 24.88%). The size of the training dataset is usually about 2 to 3 times of the size of the test dataset to increase the precision in prediction (e.g., Sinharay, 2016; Fossey, 2017).

### Instrumentation

**Figure 1:**
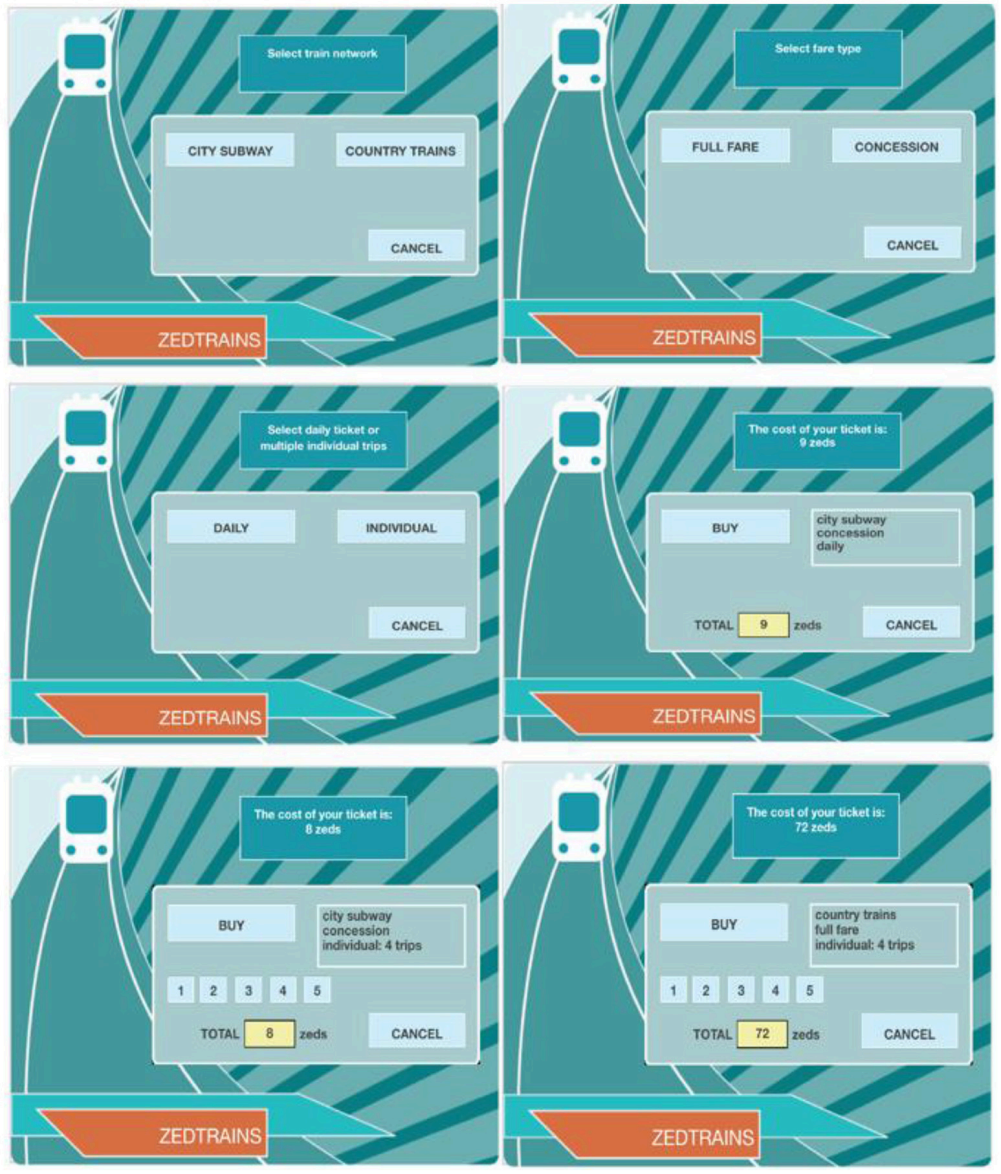
PISA 2012 problem-solving question TICKETS task2 (CP038Q01) screenshots. (For more clear view, please see http://www.oecd.org/pisa/test-2012/testquestions/question5/).

There are 42 problem-solving questions in 16 units in 2012 PISA. These items assess cognitive process in solving real-life problems in computer-based simulated scenarios (Organisation for Economic Co-operation Development, 2014). The problem-solving item, TICKETS task2 (CP038Q01), was analyzed in the current study. It is a level-5 question (there were six levels in total) that requires a higher level of exploring and understanding ability in solving this complex problem (Organisation for Economic Co-operation Development, 2014). This interactive question requires students explore and collect necessary information to make a decision. The main cognitive processes involved in this task are planning and executing. Given the problem-solving scenario, students need to come up with a plan and test it and modify it if needed. The item asks students to use their concession fare to find and buy the cheapest ticket that allows them to take 4 trips around the city on the subway within 1 day. One possible solution is to choose 4 individual concession tickets for city subway, which costs 8 zeds while the other is to choose one daily concession ticket for city subway, which costs 9 zeds. Figure 1 includes these two options. Students can always use “CANCEL” button before “BUY” to make changes. Correctly completing this task requires students to consider these two alternative solutions, then make comparisons in terms of the costs and end up choosing the cheaper one.

This item is scored polytomously with three score points, 0, 1, or 2. Students who derive only one solution and fail to compare with the other get partial credits. Students who do not come up with either of the two solutions, but rather buy the wrong ticket, get no credit on this item. For example, the last picture in Figure 1 illustrates the tickets for four individual full fare for country trains, which cost 72 zeds. “COUNTRY TRAINS” and “FULL FARE” are considered as unrelated actions because they are not the necessary actions to accomplish the task this item requires. In terms of scoring, unrelated actions are allowed as long as the students buy the correct ticket in the end and make comparisons during the action process.

### Data description

**Figure 2:**
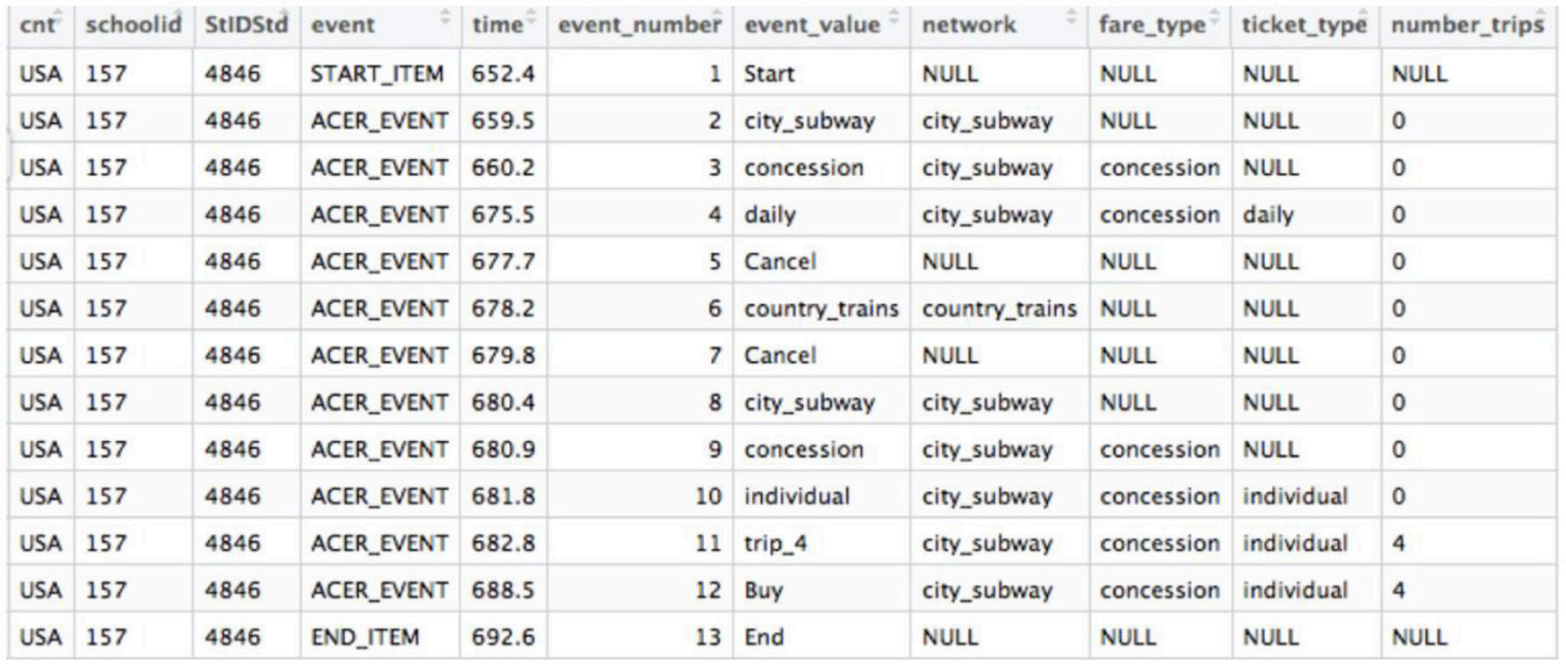
The screenshot of the log file for one student.

The PISA 2012 log file dataset for the problem-solving item was downloaded at http://www.oecd.org/pisa/pisaproducts/database-cbapisa2012.htm. The dataset consists of 4722 actions from 426 students as rows and 11 variables as columns. Eleven variables (see Figure 2) include: *cnt* indicates country, which is USA in the present study; *schoolid* and *StIDStd* indicate the unique school and student IDs, respectively; *event_number* (ranging from 1 to 47) indicates the cumulative number of actions the student took; *event_value* (see raw event_values presented in Table 1) tells the specific action the student took at one time stamp and *time* indicates the exact time stamp (in seconds) corresponding to the *event_value*. Event notifies the nature of the action (start item, end item, or actions in process). Lastly, *network, fare_type, ticket_type*, and *number_trips* all describe the current choice the student had made. The variables used were *schoolid, StIDStd, event_value* and *time*. ID variables helped to identify students, while *event_value* and *time* variables were used to generate features. The scores for all students were not provided in the log file, thus, hand coded and carefully double checked based on the scoring rule. Among the 426 students, 121 (28.4%) got full credit, 224 (52.6%) got partial credit and 81 (19.0%) did not get any credit. Full, partial, and no credit were coded as 2, 1, and 0, respectively.

**Table 1:** 15 raw event values and 36 generated features.

Event_value (15)	Start, End, city_subway, concession, full_fare, daily, Cancel, country_trains, individual, Buy, trip_1, trip_2, trip_3, trip_4, trip_5
Time features (4)	T_time, A_time, S_time, E_time
Single actions (12)	All in raw event_values except for Start, End and Buy
Two actions coded together (18)	S_city (Start → city_subway) S_country (Start → country_trains) city_full (city_subway → full_fare) city_concession (city_subway → concession) country_full (country_trains → full_fare) country_concession (country_trains → concession) concession_daily (concession → daily) concession_individual (concession → individual) full_daily (full_fare → daily) full_individual (full_fare → individual) individual_trip4 (individual → trip_4) other_cancel (other → Cancel) daily_cancel (daily → Cancel) trip4_cancel (trip_4 → Cancel) daily_buy (daily → Buy) trip4_buy (trip_4 → Buy) individual_other (individual → other) other_buy (other → Buy)
Four actions coded together (2)	city_con_ind_4 (city_subway → concession → individual → trip_4) city_con_daily_cancel (city_subway → concession → daily → Cancel)

### Feature generation and selection

#### Feature generation

Features generated can be categorized into time features and action features, as summarized in Table 1. Four Time features were created: T_time, A_time, S_time, and E_time, indicating total response time, action time spent in process, starting time spent on first action, and ending time spent on last action, respectively. It was assumed that students with different ability levels may differ in the time they read the question (starting time spent on first action), the time they spent during the response (action time spent in process), and the time they used to make final decision (ending time spent on last action). Different researchers have proposed various joint modeling approaches for both response accuracy and response times, which explain the relationship between the two (e.g., van der Linden, 2007; Bolsinova et al., 2017). Thus, the total response times are expected to differ as well.

However, in this study, action features were created by coding different lengths of adjacent action sequences together. Thus, this study generated 12 action features consisting of only one action (unigrams), 18 action features containing two ordered adjacent actions (bigrams), and 2 action features created from four sequential actions (four-grams). Further, all action sequences generated were assumed to have equal importance and no weights were assigned to each action sequence. In Table 1, “concession” is a unigram, consisting of only one action, that is, the student bought the concession fare; on the other hand, “S_city” is a bigram, consisting of two actions, which are “Start” and “city subway,” representing the student selected the city subway ticket after starting the item.

Sao Pedro et al. (2012) showed that features generated should be theoretically important to the construct to achieve better interpretability and efficiency. Following their suggestion, features were generated as the indicators of the problem-solving ability measured by this item, which is supported by the scoring rubric. For example, one action sequence consisted of four actions, which was coded as “city_con_daily_cancel,” is crucial to scoring. If the student first chose “city_subway” to tour the city, then used the student's concession fare (“concession”), looked at the price of daily pass (“daily”) next and lastly, he/she clicked “Cancel” to see the other option, this action sequence is necessary but not sufficient for a full credit.

The final recoded dataset for analysis is made up of 426 students as rows and 36 features (including 32 action sequence features and 4 time features) as columns. Scores for each student served as known labels when applying supervised learning methods. The frequency of each generated action feature was calculated for each student.

#### Feature selection

The selection of features should base on both theoretical framework and the algorithms used. As features were generated from a purely theoretical perspective in this study, no such consideration is needed in feature selection.

**Figure 3:**
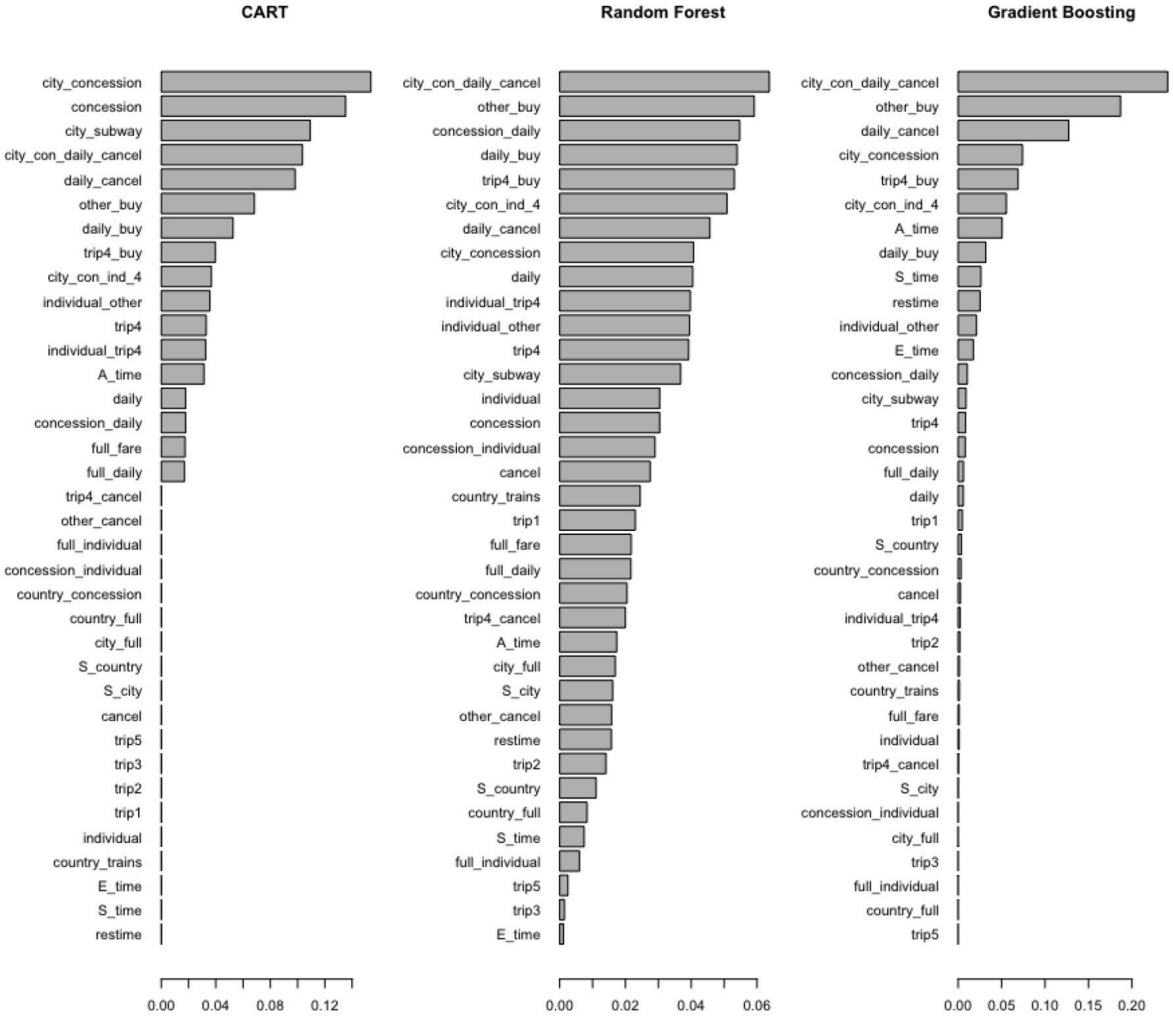
Feature importance indicated by tree-based methods.

Two other issues that need consideration are redundant variables and variables with little variance. Tree-based methods handle these two issues well and have built-in mechanisms for feature selection. The feature importance indicated by tree-based methods are shown in Figure 3. In both random forest and gradient boosting, the most important one is “city_con_daily_cancel.” The next important one is “other_buy,” which means the student did not choose trip_4 before the action “Buy.” The feature importance indicated by tree-based methods is especially helpful when selection has to be made among hundreds of features. It can help to narrow down the number of features to track, analyze, and interpret. The classification accuracy of the support vector machine (SVM) is reduced due to redundant variables. However, given the number of features (36) is relatively small in the current study, deleting highly correlated variables (ρ≥ 0.8) did not improve classification accuracy for SVM.

Clustering algorithms are affected by variables with near zero variance. Fossey (2017) and Kerr et al. (2011) discarded variables with 5 or fewer attempts in their studies. However, their data were binary and no clear-cut criterion exists for feature elimination when using cluster algorithms in the analysis of process data. In the current study, 5 features with variance no >0.09 in both training and test dataset were removed to achieve optimal classification results. Descriptive statistics for all 36 features can be found in Table A1 in Appendix A.

In summary, a full set of features (36) were retained in the tree-based methods and SVM while 31 features were selected for SOM and *k*-means after the deletion of features with little variance.

### Data mining techniques

This study demonstrates how to utilize data mining techniques to map the selected features (both action and time) to students' item performance on this problem-solving item in 2012 PISA. Given students' item scores are available in the data file, supervised learning algorithms can be trained to help classify students based on their known item performance (i.e., score category) in the training dataset while unsupervised learning algorithms categorize students into groups based on input variables without knowing their item performance. No assumptions about the data distribution are made on these data mining techniques.

Four supervised learning methods: Classification and Regression Tree (CART), gradient boosting, random forest, and SVM are explored to develop classifiers while, two unsupervised learning methods, Self-organizing Map (SOM) and *k*-means, are utilized to further examine different strategies used by students in both the same and different score categories. CART was chosen because it worked effectively in a previous study (DiCerbo and Kidwai, 2013) and is known for its quick computation and simple interpretation. However, it might not have the optimal performance compared with other methods. Furthermore, small changes in the data can change the tree structure dramatically (Kuhn, 2013). Thus, gradient boosting and random forest, which can improve the performance of trees via ensemble methods, were also used for comparison. Though SVM has not been used much in the analysis of process data yet, it has been applied as one of the most popular and flexible supervised learning techniques for other psychometric analysis such as automatic scoring (Vapnik, 1995). The two clustering algorithms, SOM and *k*-means, have been applied in the analysis of process data in log files (Stevens and Casillas, 2006; Fossey, 2017). Researchers have suggested to use more than one clustering methods to validate the clustering solutions (Xu et al., 2013). All the analyses were conducted in the software program Rstudio (RStudio Team, 2017).

### Classifier development

The general classifier building process for the supervised learning methods consists of three steps: (1) train the classifier through estimating model parameters; (2) determine the values of tuning parameters to avoid issues such as “overfitting” (i.e., the statistical model fits too closely to one dataset but fails to generalize to other datasets) and finalize the classifier; (3) calculate the accuracy of the classifier based on the test dataset. In general, training and tuning are often conducted based on the same training dataset. However, some studies may further split the training dataset into two parts, one for training while the other for tuning. Though tree-based methods are not affected by the scaling issue, training and test datasets are scaled for SVM, SOM, and *k*-means.

Given the relatively small sample size of the current dataset, training, and tuning processes were both conducted on the training dataset. Classification accuracy was evaluated with the test dataset. For the CART technique, the cost-complexity parameter (*cp*) was tuned to find the optimal tree depth using R package *rpart*. Gradient boosting was carried out using R package *gbm*. The tuning parameters for gradient boosting were the number of trees, the complexity of trees, the learning rate and the minimum number of observations in the tree's terminal nodes. Random forest was tuned over its number of predictors sampled for splitting at each node (*m*_*try*_) using R package *randomForest*. A radial basis function kernel SVM, carried out in R package *kernlab*, was tuned through two parameters: scale function σ and the cost value C, which determine the complexity of the decision boundary. After the parameters were tuned, the classifiers were trained fitting to the training dataset. 10-fold-validation was conducted for supervised learning methods in the training processes. Cross-validation is not necessary for random forest when estimating test error due to its statistical properties (Sinharay, 2016).

For the unsupervised learning methods, SOM was carried out in the R package *kohonen*. Learning rate declined from 0.05 to 0.01 over the updates from 2000 iterations. *k*-means was carried out using the *kmeans* function in the *stats* R package with 2000 iterations. Euclidian distance was used as a distance measure for both methods. The number of clusters ranged from 3 to 10. The lower bound was set to be 3 due to the three score categories in this dataset. The upper bound was set to be 10 given the relative small number of features and small sample size in the current study. The R code for the usage of both supervised and unsupervised methods can be found in Appendix B.

### Evaluation criterion

For the supervised methods, students in the test dataset are classified based on the classifier developed based on the training dataset. The performance of supervised learning techniques was evaluated in terms of classification accuracy. Outcome measures include overall accuracy, balanced accuracy, sensitivity, specificity, and Kappa. Since item scores are three categories, 0, 1, and 2, sensitivity, specificity and balanced accuracy were calculated as follows.

(1) $$\begin{array}{r} Sensitivity=\frac{True\;Positives}{True\;Positives+False\;Negatives,} \end{array}$$

(2) $$\begin{array}{r} Specificity=\frac{True\;Negatives}{True\;Negatives+False\;Positives,} \end{array}$$

(3) $$\begin{array}{r} Balanced\;Accuracy=\frac{Sensitivity\;+Specificity}{2} \end{array}$$

where sensitivity measures the ability to predict positive cases, specificity measures the ability to predict negative cases and balanced accuracy is the average of the two. Overall accuracy and Kappa were calculated for each method based on the following formula:

(4) $$\begin{array}{rcl} Overall\;Accuracy & = & \frac{True\;Positives+True\;Negatives}{Total\;Cases} \end{array}$$

(5) $$\begin{array}{rcl} Kappa & = & \frac{p_{o}-p_{e}}{1-p_{e}} \end{array}$$

where overall accuracy measures the proportion of all correct predictions. Kappa statistic is a measure of concordance for categorical data. In its formula, *p*_*o*_ is the observed proportion of agreement, *p*_*e*_ is the proportion of agreement expected by chance. The larger these five statistics are, the better classification decisions.

For the two unsupervised learning methods, the better fitting method and the number of clusters were determined for the training dataset by the following criteria:

1. Davies-Bouldin Index (DBI; Davies and Bouldin, 1979) calculated as in Equation 6, can be applied to compare the performance of multiple clustering algorithms (Fossey, 2017). The algorithm with the lower DBI is considered the better fitting one which has the higher between-cluster variance and smaller within-cluster variance.
  (6) $$\begin{array}{r} \text{DBI}=\frac{1}{k}\sum_{i=1}^{k}{\text{max}}_{i\neq j}\frac{S_{i}+S_{j}}{M_{ij}} \end{array}$$
  where *k* is the number of clusters, *S*_*i*_ and *S*_*j*_ are the average distances from the cluster center to each case in cluster *i* and cluster *j*. *M*_*ij*_ is the distance between the centers of cluster *i* and cluster *j*. Cluster *j* has the smallest between-cluster distance with cluster *i* or has the highest within-cluster variance, or both (Davies and Bouldin, 1979).
2. Kappa value (see Equation 5) is a measure of classification consistency between these two unsupervised algorithms. It is usually expected not smaller than 0.8 (Landis and Koch, 1977).
  To check the classification stability and consistency in the training dataset, the methods were repeated in the test dataset, DBI and Kappa values were computed.

## Results

The tuning and training results for the four supervised learning techniques are first reported and then the evaluation of their performance on the test datasets. Lastly, the results for the unsupervised learning methods are presented.

### Supervised learning methods

**Figure 4:**
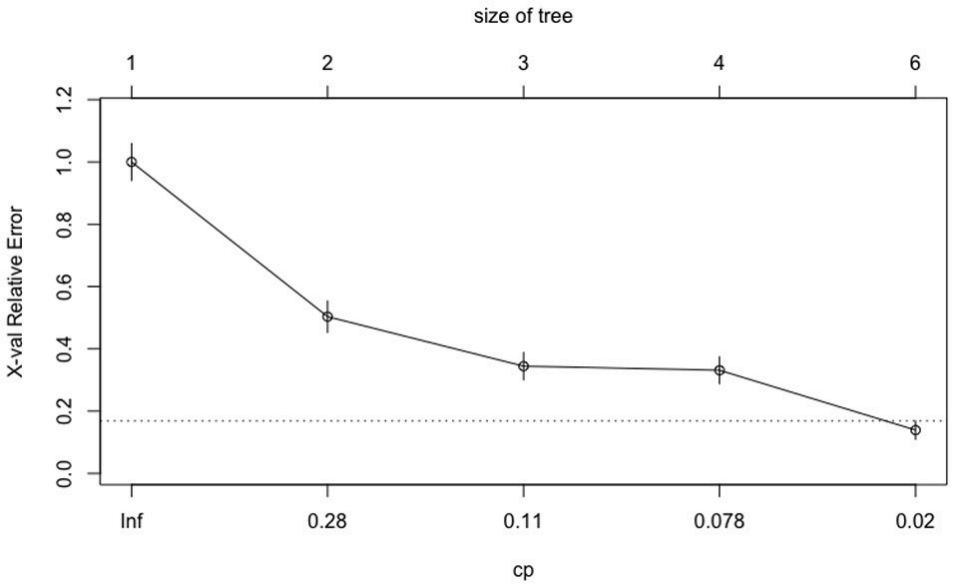
The CART tuning results for cost-complexity parameter (*cp*).

**Figure 5:**
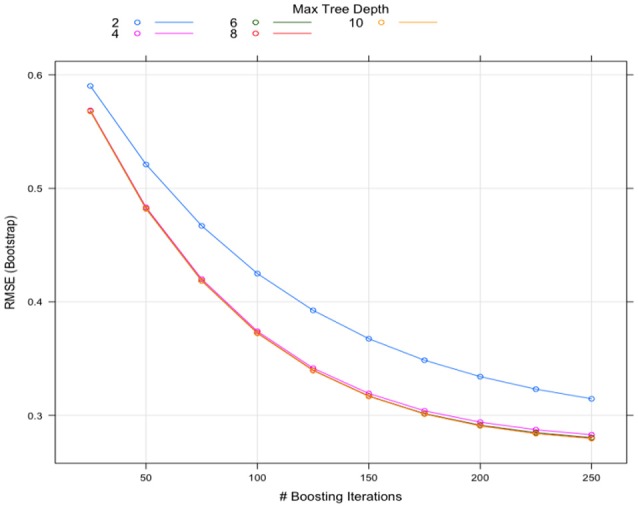
The Gradient Boosting tuning results.

**Figure 6:**
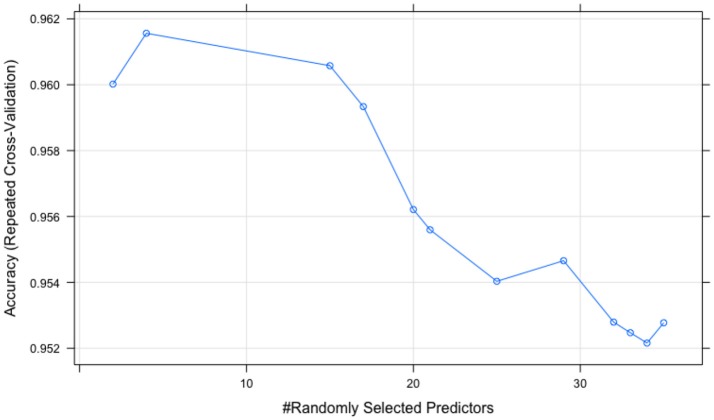
The random forest tuning results (peak point corresponds to *m*_*try*_ = 4).

The tuning processes for all the classifiers reached satisfactory results. For the CART, *cp* was set to 0.02 to achieve minimum error and the simplest tree structure (error < 0.2, number of trees < 6), as shown in Figure 4. The final tuning parameters for gradient boosting: the number of trees = 250, the depth of trees = 10, the learning rate = 0.01 and the minimum number of observations in the trees terminal nodes = 10. Figure 5 shows that when the maximum tree depth equaled 10, the RMSE was minimum as iteration reached 250 with the simplest tree structure. The number of predictors sampled for splitting at each node (*m*_*try*_) in the random forest was set to 4 to achieve the largest accuracy, as shown in Figure 6. In the SVM, the scale function σ was set to 1 and the cost value *C* set to 4 to reach the smallest training error 0.038.

The performance of the four supervised techniques was summarized in Table 2. All four methods performed satisfactorily, with almost all values larger than 0.90. The gradient boosting showed the best classification accuracy overall, exhibiting the highest Kappa and overall accuracy (Kappa = 0.94, overall accuracy = 0.96). Most of their subclass specificity and balanced accuracy values also ranked top, with only sensitivity for score = 0, specificity for score = 1 and balanced accuracy for score = 0 smaller than those from SVM. SVM, random forest, and CART performed similarly well, all with a slightly smaller Kappa and overall accuracy values (Kappa = 0.92, overall accuracy = 0.95).

**Table 2:** Average of accuracy measures of the scores.

**Method**	**R package**	**Kappa**	**Overall accuracy**	**Sensitivity**			**Specificity**			**Balanced accuracy**		
				**0**	**1**	**2**	**0**	**1**	**2**	**0**	**1**	**2**
CART	rpart	0.92	0.95	0.89	0.97	0.97	0.98	0.96	0.99	0.93	0.96	0.98
Random Forest	randomForest	0.92	0.95	0.89	0.95	10.0	0.99	0.96	0.97	0.94	0.95	0.99
Gradient Boosting	gbm	0.94	0.96	0.89	0.97	10.0	0.99	0.96	0.99	0.94	0.96	0.99
Support Vector Machine	kernlab	0.92	0.95	0.94	0.93	10.0	0.98	0.98	0.97	0.96	0.96	0.99

**Figure 7:**
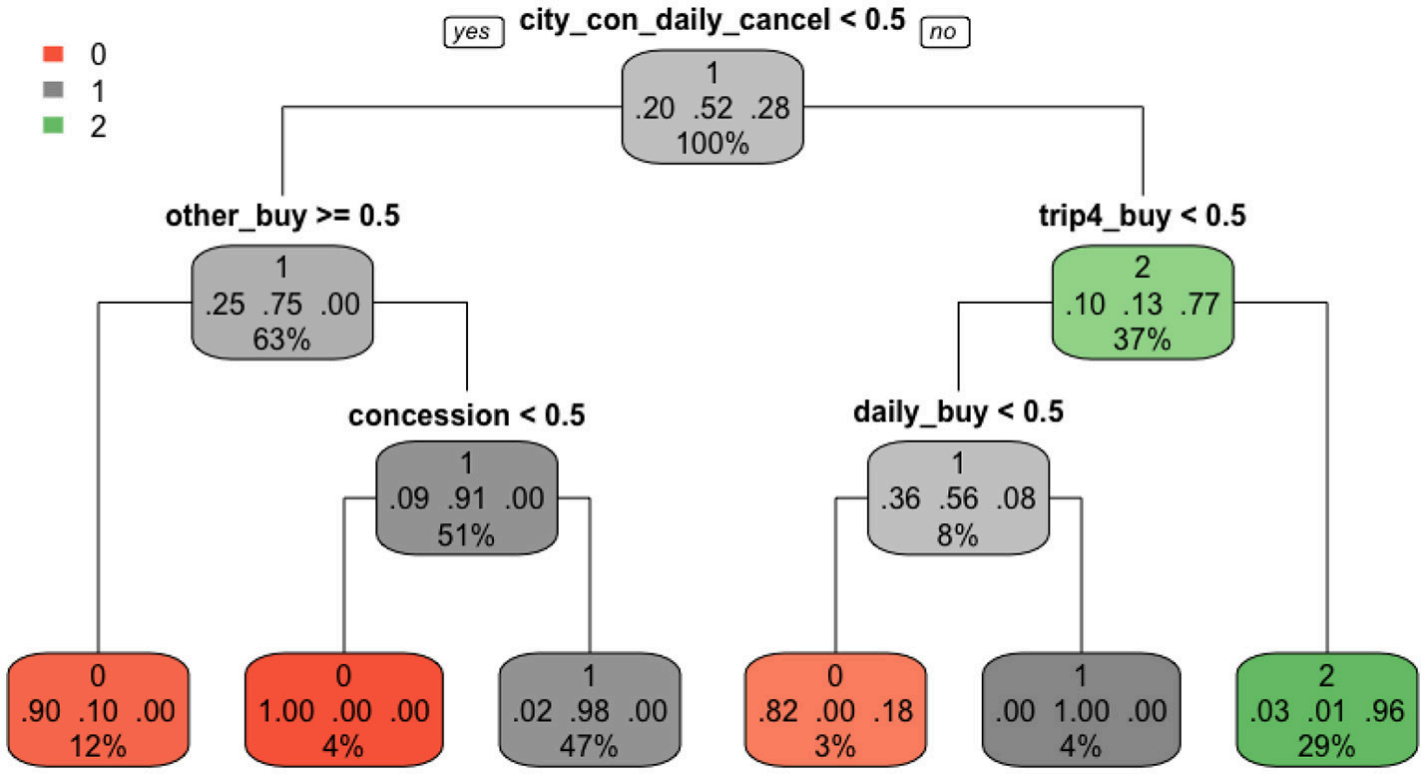
The CART classification.

Among the four supervised methods, the single tree structure from CART built from the training dataset is the easiest to interpret and plotted in Figure 7. Three colors represent three score categories: red (no credit), gray (partial credit), and green (full credit). The darker the color is, the more confident the predicted score is in that node, the more precise the classification is. In each node, we can see three lines of numbers. The first line indicates the main score category in that node. The second line represents the proportions of each score category, in the order of scores of 0, 1, and 2. The third line is the percentage of students falling into that node. CART has a built-in characteristic to automatically choose useful features. As shown in Figure 7, only five nodes (features), “city_con_daily_cancel,” “other_buy,” “trip4_buy,” “concession,” and “daily_buy,” were used in branching before the final stage. In each branch, if the student performs the action (>0.5), he/she is classified to the right, otherwise, to the left. As a result, students with a full credit were branched into one class, in which 96% truly belonged to this class and accounted for 29% of the total data points. Students who earned a partial credit were partitioned into two classes, one purely consisted of students in this group and the other consisted of 98% students who truly got partial credit. For the no credit group, students were classified into three classes, one purely consisted of students in this group and the other two classes included 10 and 18% students from other categories. One major benefit from this plot is that we can clearly tell the specific action sequences that led students into each class.

### Unsupervised learning methods

**Figure 8:**
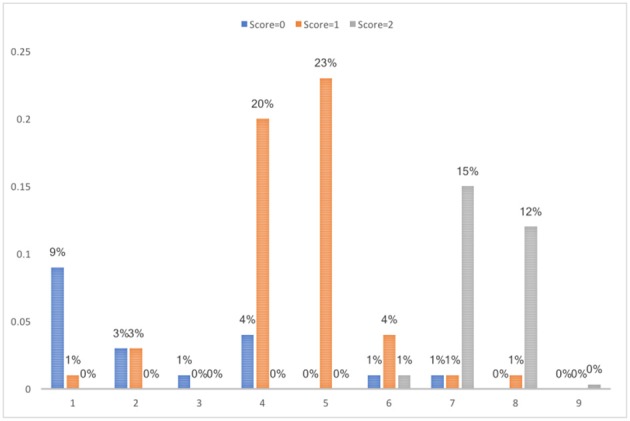
Percentage in each score category in the final SOM clustering solution with 9 clusters from the training dataset.

As shown in Table 3, the candidates for the best clustering solution from the training dataset were *k*-means with 5 clusters (DBI = 0.19, kappa = 0.84) and SOM with 9 clusters (DBI = 0.25, kappa = 0.96), which satisfied the criterion of a smaller DBI value and kappa value ≥ 0.8. When validated with the test dataset, the DBI values for *k*-means and SOM all increased. It could be caused by the smaller sample size of the test dataset. Due to the low kappa value for the 5-cluster solution in the validation sample, the final decision on the clustering solution was SOM with 9 clusters. The percentage of students in each score category in each cluster is presented in Figure 8. The cluster analysis results obtained based on both SOM and *k*-means can be found in Table A2 in Appendix A.

**Table 3:** Clustering Algorithms' Fit (DBI) and Agreement (Cohen's Kappa).

	**Training dataset (*****n*** = **320)**			**Test dataset (*****n*** = **106)**		
**Number of clusters**	**DBI**		**Kappa**	**DBI**		**Kappa**
	***k*-means**	**SOM**		***k*-means**	**SOM**
3	1.427	1.54	0.037	1.741	1.696	0.900
4	1.792	1.447	0.061	1.444	1.178	0.078
5	0.188**	1.296	**0.843**	1.098	1.133	0.320**
6	1.448	1.087	0.934	1.057	1.171	0.390
7	1.413	1.023	0.835	1.177	0.920	0.891
8	0.198	1.057	0.753	1.063	1.034	0.894
9	1.099	0.249*	**0.959**	1.288	0.979	0.831
10	1.442	0.251	0.884	1.288	0.816	0.627

** *Best fitting solution with the training dataset but lower Kappa value with the test dataset, indicating the disagreement between k-means and SOM*.

* *Final chosen solution. Bold values indicate potential final clustering solution and are discussed in the text*.

To interpret, label and group the resulting clusters, it is necessary to examine and generalize the students' features and the strategy pattern in each of the cluster. In alignment with the scoring rubrics and ease of interpretation, the nine clusters identified in the training dataset are grouped into five classes and interpreted as follows.

1. Incorrect (cluster1): students bought neither individual tickets for 4 trips nor a daily ticket.
2. Partially correct (cluster 4–5): students bought either individual tickets for 4 trips or a daily ticket but did not compare the prices.
3. Correct (cluster 7 and 8): students did compare the prices between individual tickets and a daily ticket and chose to buy the cheaper one (individual tickets for 4 trips).
4. Unnecessary actions (cluster 2, 3, and 6): students tried options not required by the question, e.g., country train ticket, other number of individual ticket.
5. Outlier (cluster 9): the student made too many attempts and is identified as an outlier.

Such grouping and labeling can help researchers better understand the common strategies used by students in each score category. It also helps to identify errors students made and can be a good source of feedback to students. For those students mislabeled above, they share the major characteristics in the cluster. For example, 4% students who got no credit in cluster 4 in the training dataset bought daily ticket for the city subway without comparing the prices, but they bought the full fare instead of using student's concession fare. These students are different from those in cluster 1 who bought neither daily tickets nor individual tickets for 4 trips. Thus, students in the same score category were classified into different clusters, indicating that they made different errors or took different actions during the problem-solving process. In summary, though students in the same score category generally share the actions they took, they can also follow distinct problem-solving processes. Students in different score categories can also share similar problem-solving process.

## Summary and discussions

This study analyzed the process data in the log file from one of the 2012 PISA problem-solving items using data mining techniques. The data mining methods used, including CART, gradient boosting, random forest, SVM, SOM, and *k*-means, yielded satisfactory results with this dataset. The three major purposes of the current study were summarized as follows.

First, to demonstrate the analysis of process data using both supervised and unsupervised techniques, concrete steps in feature generation, feature selection, classifier development and outcome evaluation were presented in the current study. Among all steps, feature generation was the most crucial one because the quality of features determines the classification results to a large extent. Good features should be created based on a thorough understanding of the item scoring procedure and the construct. Key action sequences that can distinguish correct and incorrect answers served as features with good performance. Unexpectedly, time features, including total response time and its pieces, did not turn out to be important features for classification. This means that considerable variance of response time existed in each score group and the differences in response time distributions among the groups was not large enough to clearly distinguish the groups (see Figure A1 in Appendix A). This study generated features based on theoretical beliefs about the construct measured and used students as the unit of analysis. The data could be structured in other ways according to different research questions. For example, instead of using students as the unit of analysis, the attempts students made can be used as rows and actions as columns, then the attempts can be classified instead of people. Fossey (2017) included a detailed tutorial on clustering algorithms with such data structure in a game-based assessment.

Second, to evaluate classification consistency of these frequently used data mining techniques, the current study compared four supervised techniques with different properties, namely, CART, gradient boosting, random forest, and SVM. All four methods achieved satisfactory classification accuracy based on various outcome measures, with gradient boosting showing slightly better overall accuracy and Kappa value. In general, easy interpretability and graphical visualization are the major advantages of trees. Trees also deal with noisy and incomplete data well (James et al., 2013). However, the trees are easily influenced by even small changes in the data due to its hierarchical splitting structure (Hastie et al., 2009). SVM, on the contrary, generalizes well because once the hyperplane is found, small changes to data cannot greatly affect the hyperplane (James et al., 2013). Given the specific dataset in the current study, even the CART method worked very well. In addition, the CART method can be easily understood and provided enough information about the detailed classifications between and within each score category. Thus, based on the results in the current study, the CART method is sufficient for future studies on similar datasets. Unsupervised learning algorithms, SOM and *k*-means, also showed convergent clustering results based on DBI and Kappa values. In the final clustering solution, students were grouped into 9 clusters, revealing specific problem-solving processes they went through.

Third, supervised and unsupervised learning methods serve to answer different research questions. Supervised learning methods can be used to train the algorithm to predict memberships in the future data, like automatic scoring. Unsupervised methods can reveal the problem-solving strategy patterns and further differentiate students in the same score category. This is especially helpful for formative purposes. Students can be provided with more detailed and individualized diagnostic reports. Teachers can better understand students' strengths and weaknesses, and adjust instructions in the classroom accordingly or provide more targeted tutoring to specific students. In addition, it is necessary to check any indication for cheating behavior in the misclassified or outlier cases from both types of data mining methods. For example, students answered the item correctly within an extremely short amount of time can imply item compromise.

This study has its own limitations. Other data mining methods, such as other decision trees algorithms and clustering algorithms, are worth of investigation. However, the procedure demonstrated in this study can be easily generalized to other algorithms. In addition, the six methods were compared based on the same set of data rather than data under various conditions. Therefore, the generalization of the current study is limited due to factors such as sample size and number of features. Future studies can use a larger sample size and extract more features from more complicated assessment scenarios. Lastly, the current study focuses on only one item for the didactic purpose. In the future study, process data for more items can be analyzed simultaneously to get a comprehensive picture of the students.

To sum up, the selection of data mining techniques for the analysis of process data in assessment depends on the purpose of the analysis and the data structure. Supervised and unsupervised techniques essentially serve different purposes for data mining with the former as a confirmatory approach while the latter as an exploratory approach.

## Author contributions

XQ as the first author, conducted the major part of study design, data analysis and manuscript writing. HJ as the second author, participated in the formulation and refinement of the study design and provided crucial guidance in the statistical analysis and manuscript composition.

### Conflict of interest statement

The authors declare that the research was conducted in the absence of any commercial or financial relationships that could be construed as a potential conflict of interest.
