# Supplementary material for: Data Mining Techniques in Analyzing Process Data: A Didactic
Source: Front Psychol. 2018 Nov 23;9:2231. doi: 10.3389/fpsyg.2018.02231 (PMC6265513; doi:10.3389/fpsyg.2018.02231)
Supplement: Supplementary file 1 [file Table_1.pdf]

Appendix A

Table A1

Descriptive Statistics for the 36 Features

Features	Training data				Test data			
	Mean	SD	No attempt	Attempt	Mean	SD	No attempt	Attempt
T_time	52.5	22.0			53.6	24.3		
A_time	30.4	17.8			32.2	20.1		
S_time	17.4	11.4			16.7	9.9		
E_time	4.9	5.0			5.1	5.0		
city_subway	1.7	1.2	23	297	1.7	1.3	7	99
country_trains	0.3	0.7	251	69	0.2	0.6	90	16
full_fare	0.1	0.5	284	36	0.1	0.3	95	11
concession	1.8	1.2	17	303	1.8	1.4	4	102
daily	0.8	0.8	118	202	0.9	0.8	30	76
individual	1.0	0.9	82	238	1.0	0.9	32	74
trip1	0.2	0.7	267	53	0.2	0.8	91	15
trip2	0.1	0.3	295	25	0.1	0.4	96	10
trip3	0.1	0.3	305	15	0.1	0.4	100	6
trip4	0.9	1.0	134	186	0.9	1.1	46	60
trip5	0.1	0.3	307	13	0.1	0.5	99	7
cancel	1.0	1.6	176	144	1.0	1.6	58	48
**S_city**	**0.9**	**0.3**	**32**	**288**	**0.9**	**0.3**	**8**	**98**
**S_country**	**0.1**	**0.3**	**291**	**29**	**0.1**	**0.2**	**99**	**7**
city_full	0.1	0.4	295	25	0.1	0.3	95	11
city_concession	1.6	1.1	31	289	1.6	1.2	10	96
**country_full**	**0.1**	**0.3**	**306**	**14**	**0.0**	**0.0**	**106**	**0**
country_ concession	0.2	0.6	277	43	0.2	0.5	94	12
concession_daily	0.7	0.7	127	193	0.8	0.7	33	73
concession_ individual	1.0	0.8	89	231	0.9	0.9	33	73
**full_daily**	**0.1**	**0.3**	**303**	**17**	**0.0**	**0.2**	**102**	**4**
**full_individual**	**0.0**	**0.2**	**309**	**11**	**0.0**	**0.1**	**104**	**2**
individual_trip4	0.7	0.9	146	174	0.7	0.9	54	52
other_cancel	0.3	0.8	258	62	0.3	0.7	87	19
daily_cancel	0.5	0.8	194	126	0.5	0.8	63	43
trip4_cancel	0.2	0.6	267	53	0.2	0.6	89	17
daily_buy	0.3	0.5	229	91	0.4	0.5	68	38
trip4_buy	0.5	0.5	145	175	0.5	0.5	54	52
individual_other	0.3	0.6	235	85	0.3	0.5	82	24
other_buy	0.2	0.4	269	51	0.1	0.4	91	15
city_con_daily_cancel	0.4	0.6	202	118	0.5	0.6	64	42
city_con_ind_4	0.6	0.7	152	168	0.7	0.8	54	52

Note. SD = Standard Deviation; No attempt = Count of action sequences that no students took; Attempt = Count of action sequences that were taken by the students; Bolded features were those removed in the cluster analysis due to low variance (≤ .09) in both training and test data.

Table A2

Proportion of Different Classes Obtained from SOM and *k*-means

Cluster	SOM			*k*-means		
	Score = 0	Score = 1	Score = 2	Score = 0	Score = 1	Score = 2
1	9.4%	0.6%	0.0%	4.1%	5.3%	0.0%
2	2.5%	2.8%	0.0%	3.1%	5.0%	0.9%
3	1.3%	0.3%	0.0%	0.3%	0.0%	0.0%
4	4.1%	20.3%	0.0%	3.8%	12.2%	11.3%
5	0.0%	22.5%	0.0%	5.9%	21.9%	2.8%
6	0.6%	3.8%	0.9%	0.6%	2.2%	2.5%
7	0.6%	1.3%	15.3%	1.3%	4.4%	10.0%
8	0.3%	1.3%	0.0%	0.6%	0.6%	0.9%
9	0.0%	0.0%	0.3%	0.0%	0.3%	0.0%

Note. SOM = Self-organizing Map.

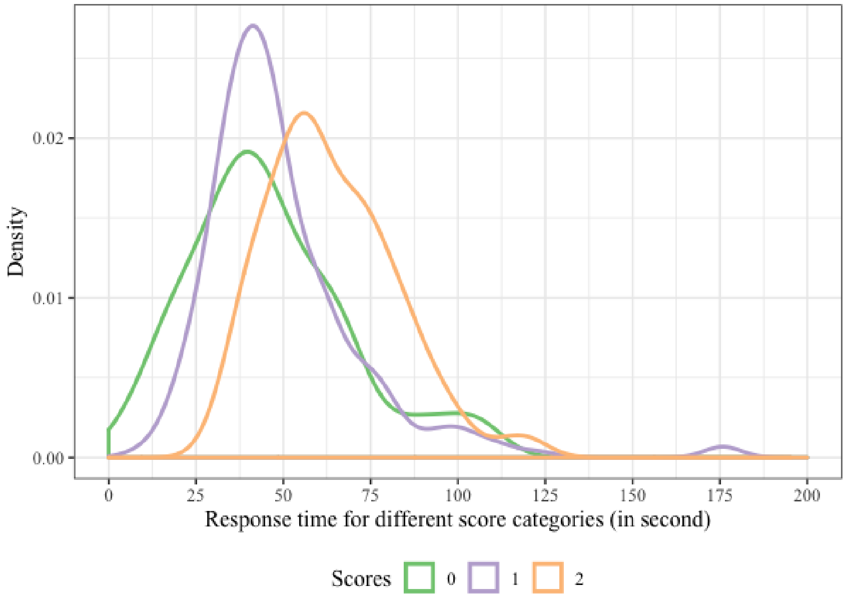

Figure A1. Density Plot of the Response Times from the US Sample

Appendix B: R Script for Data Mining Techniques

```
#######CART#########
library(rpart)
# rpart automatically uses 10-fold cross-validation to prune the tree
model<-rpart(labels ~ .,data=traindata, method = "class")
print(model)
plotcp(model)
#visualize the rpart model
library(rpart.plot)
rpart.plot(model,type=1)
##testing
predmodel<-predict(model,newdata = testdata,cp=.02,type="class")

########Gradient Boosting######
## tuning
library(caret)
bootCtrl <- trainControl(number = 200)
gbmGrid <- expand.grid(interaction.depth = (1:5) * 2,
                    n.trees = (1:10)*25, shrinkage shrinkage =
seq(0.001,0.01,0.1),n.minobsinnode=c(5,10,15))
gbmFit <- train(traindata[,c(1:36)], traindata$labels,
             method = "gbm", trControl = bootCtrl, verbose = FALSE,
             bag.fraction = 0.5, tuneGrid = gbmGrid)
plot(gbmFit)
gbmFit$bestTune

##training
#for binary classification problems, distribution= “bernoulli”
library(gbm)
gbm<- gbm(labels~.,
data=traindata,distribution="multinomial",n.trees=250,shrinkage=.01,n.minobsinnode =
10,interaction.depth = 10, cv.folds = 10)

##testing
predBoost<-predict(gbm, newdata=testdata, type="response")

#######Random Forrest#########
#tuning
control <- trainControl(method="repeatedcv", number=10, repeats=10, search="random")
rf_random <- train(labels~., data=traindata, method="rf", metric="Accuracy", tuneLength=15,
trControl=control)
print(rf_random)
plot(rf_random)

#training
```

```
library(randomForest)
RandomForest<-randomForest(labels~., data=traindata, mtry=4)
##testing
predRF<-predict(RandomForest,newdata=testdata,type="class")

#######SVM#########
#tuning
library(e1071)
tune.out=tune(svm , labels~., data=train_scaled, kernel="radial",
              ranges =list(cost=c(0.1 ,1 ,10 ,100 ,1000),
                           gamma=c(0.5,1,2,3,4) ))
summary (tune.out)

#training
library(kernlab)
svmmodel<-
ksvm(labels~.,data=train_scaled,kernel="rbfdot",C=1,cross=10,cprob.model=TRUE)
print(svmmodel)
#testing
svmPred <-predict(svmmodel,test_scaled,type="response")


######SOM#####
#k=number of clusters
library(kohonen)
som=som(as.matrix(clustertrain), grid = somgrid(k, 1,
                  "hexagonal"),rlen=2000,alpha=c(.05,.01))

######k-means#####
library(stat)
kmeans=kmeans(clustertrain,centers=(k), nstart=2000)
```
